# Fabrication and Optical Characterization of VO_2_-Based Thin Films Deposited on Practical Float Glass by Magnetron Sputtering and Professional Annealing

**DOI:** 10.3390/ma15092990

**Published:** 2022-04-20

**Authors:** Xinhong Chu, Qiusheng Xie, Xiaoming Zhang, Bingfeng Guo, Jianqing Liao, Xiujian Zhao

**Affiliations:** 1 College of Physics Science and Engineering Technology, Yichun University, Yichun 336000, China; lr@jxycu.edu.cn (Q.X.); zhangxm@jxycu.edu.cn (X.Z.); guobingfeng@jxycu.edu.cn (B.G.); jq-liao@jxycu.edu.cn (J.L.); 2 State Key Laboratory of Silicate Materials for Architectures, Wuhan University of Technology, Wuhan 430070, China

**Keywords:** VO_2_, sputtering, annealing, float glass, W doping, oxygen vacancy, Na^+^ ions

## Abstract

In this paper, VO_2_ thin films with good optical properties are fabricated on practical float glass by magnetron sputtering and a professional annealing method. The near-infrared switching efficiency (NIRSE) of the prepared film reaches 39% (@2000 nm), and its near-infrared energy modulation ability (ΔT_ir_) reaches 10.9% (780–2500 nm). Further, the highest integral visible transmittance T_lum_ is 63%. The proposed method exhibits good reproducibility and does not cause any heat damage to the magnetron sputtering machine. The crystalline structure of the VO_2_ film is characterized by X-ray diffraction (XRD). The lattice planes (011) and (−211) grow preferentially (JCPDS 65-2358), and a large number of NaV_2_O_5_ crystals are detected simultaneously. The microstructures are characterized by scanning electron microscopy (SEM), and a large number of long sheet crystals are identified. The phase transition temperature is significantly reduced by an appropriate W doping concentration (T_c_ = 29 °C), whereas excessive W doping causes distortion of the thermal hysteresis loop and a reduction in the NIRSE. Oxygen vacancies are created by low pressure annealing, due to which the phase transition temperature of VO_2_ film decreases by 8 °C. The addition of an intermediate SiO_2_ layer can prevent the diffusion of Na^+^ ions and affect the preparation process of the VO_2_ thin film.

## 1. Introduction

Vanadium dioxide is a typical thermochromic smart material, which was discovered by Morin in 1959 [1]. Below 68 °C, it is a monoclinic semiconductor, while above 68 °C, it becomes a tetragonal conductor. Near the critical point, the electrical and optical properties of VO_2_ change by 2–5 orders of magnitude, and the infrared transmittance can change by 40–60% [1,2,3,4]. Therefore, VO_2_ has garnered considerable research attention for preparing smart windows [5,6], optical switches [7], biomedical sensors [8], relaxation oscillator [9], and so on.

Magnetron sputtering [10,11], chemical vapor deposition (CVD) [12], sol-gel processing [4,13], pulsed laser deposition [14], and molecular beam epitaxy [15] are often used to prepare VO_2_ films. Among these methods, magnetron sputtering is the most widely used method owing to its advantages of controllable film thickness, high speed, low temperature, good adhesion, high production efficiency, environmental friendliness, and great industrial potential, and it can be used to prepare compact, homogeneous, and stable films. However, it faces the problems of complex process, difficult operation, and poor reproducibility in preparing VO_2_ thin films. Therefore, Gurvitch et al. [16] and Luo et al. [17] firstly used magnetron sputtering to prepare metal V film and then utilized in situ oxidative annealing to prepare VO_2_ thin films, which exhibited good repeatability. However, the in-situ annealing operation at high temperature (above 300–500 °C) can damage the magnetron sputtering equipment and shorten its working life. Liu et al. [18], Xu et al. [19], and Ba et al. [20] deposited V metal films on glass and sapphire by magnetron sputtering or ion-assisted sputtering technology and then obtained VO_2_ films by oxidative annealing in pure oxygen atmosphere or air atmosphere in a furnace. This two-step technique was beneficial to improve the reproducibility and protect the sputtering equipment, but they did no further works in studies of substrates, visible transmittance improvement, doping, oxygen vacancies, and so on. Silicon [21], quartz [10,22], sapphire [10,23], and other materials are often used as substrates for depositing VO_2_ film to achieve better phase transition performance. However, these substrates are expensive, difficult to obtain, and inconsistent with the actual glass used in windows and doors. Float glass (Na-Ca-Si ternary glass, Na~13.8%, Ca~8.6%, Si~72.8%, others~4.8%) is the most widely used practical glass in modern buildings, so it must be explored as a substrate for VO_2_ to be applied in smart windows. Presently, the most vital problem of float glass is that small Na^+^ ions can easily diffuse into the VO_2_ film, which results in poor thermochromic properties [24]. VO_2_ thin films have been prepared on Si_3_N_4_ [25], TiO_2_ [26], ZnO [27], SnO_2_ [28], and other buffer layers, but the preparation methods and properties were quite different. For application in smart windows, the VO_2_ film’s visible transmittance should be above 60%, and the key performance of the transition temperature must be reduced to a practical range of 25–30 °C. Tungsten doping and oxygen vacancy production have been considered to be effective methods [29,30,31], but the mechanisms of tungsten doping for reducing the phase transition temperature and the equivalent effect of oxygen vacancy are still not clear.

In this study, a two-step method of magnetron sputtering and professional annealing with good reproducibility and no heat damage to the sputtering machine was successfully utilized to deposit VO_2_-based thermochromic thin films on a practical float glass, including intrinsic VO_2_ thin films, VO_2_ thin films with intermediate SiO_2_ layers, and W-doped VO_2_ thin films. Further, the annealing control, visible transmittance adjustment, the effect of 100-nm-thick intermediate SiO_2_ layer on preventing the diffusion of Na^+^ ions, and the equivalent action of W dopant and O vacancy were investigated.

## 2. Materials and Methods

### 2.1. Preparation of V-Based Metal Films by Magnetron Sputtering

The practical float glass used in this study was SAIL BRAND 7101 with a thickness of 1 mm. The magnetron sputtering machine used was ADV-ZKS-0350 (Aitmet Vacuum Machinery Co., Ltd., Foshan, Guangdong, China). The float glass (hereafter referred to as glass) was cut into a size of 25.4 mm × 38.1 mm, then ultrasonically cleaned in acetone and anhydrous ethanol for 30 min, and subsequently dried off for later use. The sputtering vacuum chamber was thoroughly cleaned, and then the V metal target (φ: 56 mm, 99.9% purity) and glass substrate were installed. The chamber was vacuumized to 3.0 × 10^−3^ Pa, and then Ar gas (99.99% purity) was passed through it to achieve a pressure of 1 Pa. DC magnetron sputtering was utilized to deposit the V-based metal films at room temperature (20 °C). The impurity atoms on the target surface were removed by pre-sputtering for 10 min, and then the baffle was opened to deposit films at a power of 77 W. The sputtering time was controlled to 5, 7.5, and 10 min to obtain V metal films with thicknesses of 30, 45, and 60 nm (calculated by statistical average deposition rate), respectively. The main process parameters are shown in Table 1, and the quartz glass is a contrast substrate.

**Figure 1:**
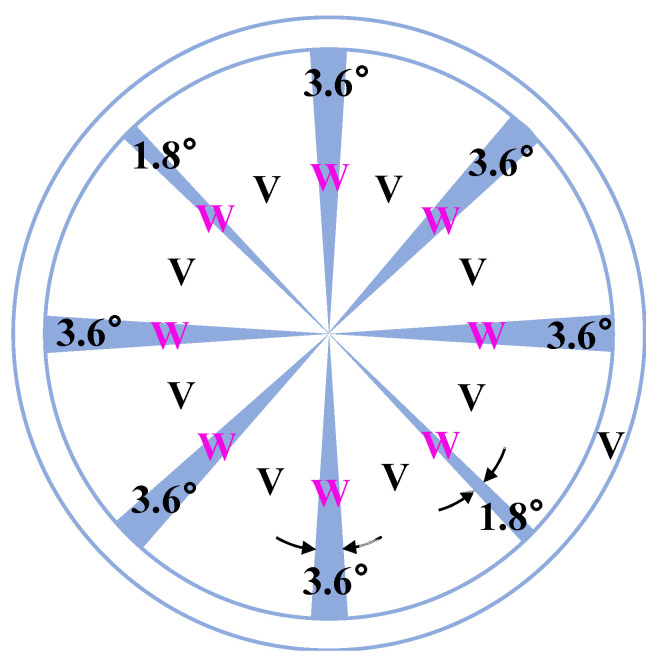
Schematic of the tungsten-vanadium inlaid target (when W bars are fully embedded, doping ratio-max: 7%). (The blue bar is W. One 1.8° W bar represents 0.5% doping ratio by area, and one 3.6° W bar represents 1.0% doping ratio by area; Increase the number of W bars can get different W doping concentrations, and the maximum can reach 7%).

W/V inlaid target (φ: 56 mm, W purity: 99.95%, V purity: 99.9%, Figure 1) and DC magnetron sputtering technology were applied to execute W doping in an appropriate ratio (0–1.0%, the ratio of the area of W bars to the total area of the target). According to the sputtering yield, sputtering area, and atomic concentration, the W dopant ratio by mol can be calculated according to the area ratio of W bars. The W dopant’s ratios by area of 0%, 0.5%, and 1% correspond to the ratios by mol of 0%, 2.83%, and 5.53%, respectively. For convenience, the W dopant ratio in this paper represents the area ratio unless otherwise specified. The sputtering time for the W:V metal film was 7.5 min, and the thickness was approximately 45 nm. The main process parameters are shown in Table 1.

RF magnetron sputtering technology was used to deposit the SiO_2_ layer on the glass for blocking the sodium ion diffusion. The main process parameters are shown in Table 2. Then, 30 nm V metal film was spread on the intermediate SiO_2_ layer (Table 1).

### 2.2. Preparation of VO_2_-Based Films by Professional Annealing

The deposited V-based metal films (V, W:V, V/SiO_2_) were placed in a vacuum tube furnace (ECFK-6-14), vacuumized to a needed low pressure, heated to 400 °C at 5 °C/min, annealed in low pressure air atmosphere for 1 h, and then naturally cooled to room temperature to obtain VO_2_-based films. The main process parameters are shown in Table 3, and the quartz glass is a contrast substrate.

### 2.3. Testing and Characterization

The transmittance of the film was examined using ultraviolet-visible-infrared spectrophotometry (UV-3600 MPC 3100, Shimadsu, Kyoto, Japan), where the testing wavelength range was 250–2500 nm, the step length was 1 nm, and the testing temperature was 20 and 90 °C. The heating attachment was a small numerical control furnace with a temperature error of ±1 °C. The near-infrared switching efficiency (NIRSE) is defined as the difference between the transmittance at low temperature and the transmittance at high temperature at 2000 nm. The thermal hysteresis loop was drawn using the transmittance curves at different temperatures in the range of 20–90 °C with an interval of 5 °C both in the heating and cooling process. Further, each temperature point was held for at least 5 min before obtaining the transmittance curve. Moreover, a standard VO_2_ film sample (quartz glass substrate, NIRSE 50%, i.e., the VO_2_ film by “30 nm V/quartz glass” in Table 3) was used to complete the transmittance measurement in whole wavelength 250–2500 nm at any two temperature points, e.g., 20 °C and 40 °C, to check the sameness and uniformity of the transmittance curve before every loop test. To obtain the thermal hysteresis loop of the VO_2_ film, the transmittance values at 2000 nm in each curve were considered as the *Y*-axis, and temperature points were taken for the *X*-axis.

The phase composition of the as-prepared VO_2_-based films were analyzed by X-ray diffraction (XRD; PANalytical Empyrean, PANalytical B.V., Almelo, Netherlands; Cu target, λ = 1.540598 Å, acceleration voltage = 40 kV, and current = 10 mA). The micro morphologies of these films were investigated by field emission scanning electron microscopy (FESEM; Hitachi S-4800, Hitachi, Tokyo, Japan). The oxygen vacancies of related films were investigated by X-ray photoelectron spectroscopy (XPS; VG Mutilab 2000, Thermo Electron Co., Bedford, MA, USA; Al Kα, hν = 1486.6 eV, resolution 0.47 eV).

### 2.4. Data Treatment

According to the transmittance curve and Equation (1), the visible spectral transmittance can be obtained [32], which is also known as the integrated visual photopic transmission (IVPT, denoted by *T_lum_*). This kind of transmittance takes the people’s visual perception into account and is more suitable for the daylighting evaluation of green buildings. It is calculated as follows:

(1) $$T_{lum}=\frac{\int_{380}^{780}D_{\lambda }V(\lambda )T(\lambda )d\lambda }{\int_{380}^{780}D_{\lambda }V(\lambda )d\lambda }$$

where *D_λ_* is the relative spectral distribution of illuminant D65, *V*(*λ*) is the spectral luminous efficiency for photopic vision, and *T*(*λ*) is the visible transmittance. Further, the values of *D_λ_*·*V*(*λ*)·Δ*λ* were obtained from the international standard ISO 9095-2003.

According to the transmission curve and Equation (2), the near-infrared spectral transmittance (*T_ir_*) can be calculated [32]. The regulation ability to near-infrared light (Δ*T_ir_*) is expressed as the difference between *T_ir_* at high temperature and T_ir_ at low temperature. *T_ir_* is calculated as follows:

(2) $$T_{ir}=\frac{\int_{780}^{2500}S_{\lambda }T(\lambda )d\lambda }{\int_{780}^{2500}S_{\lambda }d\lambda }$$

where *S_λ_* is the relative spectral distribution of solar radiation, *T*(*λ*) is the near-infrared transmittance, and the values of *S_λ_*·Δ*λ* were obtained from the international standard ISO 9095-2003.

## 3. Results

### 3.1. Transmittance and Microstructure of VO_2_ Film Based on the Optimization of Thickness and Pressure

**Figure 2:**
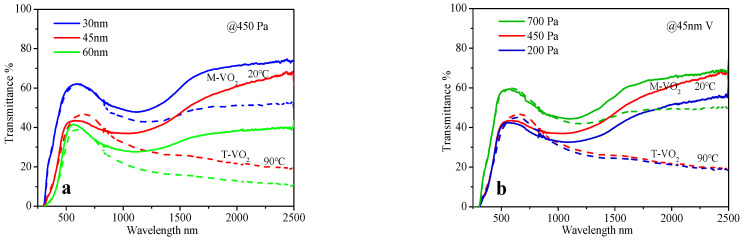
(**a**) Transmittance of VO_2_ films prepared by professional annealing of V metal films with different thicknesses at 450 Pa. (**b**) Transmittance of VO_2_ films prepared by professional annealing of 45 nm V films at different pressures; all at 400 °C for 1 h; solid line: 20 °C (monoclinic M-VO_2_), dashed line: 90 °C (tetragonal T-VO_2_).

Figure 2a shows that at the annealing pressure of 450 Pa, the NIRSE (39%) of the VO_2_ film prepared by the 45 nm V metal film is higher than that prepared by the 30 nm, 60 nm V metal films (20%, 26%). This indicates that the optimal thickness of the V film under this condition is nearly 45 nm. Furthermore, Figure 2b shows that for the 45 nm V metal film, the NIRSE (39%) of VO_2_ film prepared at the annealing pressure of 450 Pa is higher than that at 200 and 700 Pa (16%, 30%). This suggests that the optimal annealing pressure of the V film under this condition is approximately 450 Pa. When the thickness and annealing pressure of the VO_2_ film are optimized, the NIRSE is enhanced, and the values of △T_ir_ and T_lum_ change accordingly. Generally, as NIRSE increases, ΔT_ir_ increases. When the thickness of the V metal film decreases or the annealing pressure increases, T_lum_ increases, and vice versa, as illustrated in Table 4.

**Figure 3:**
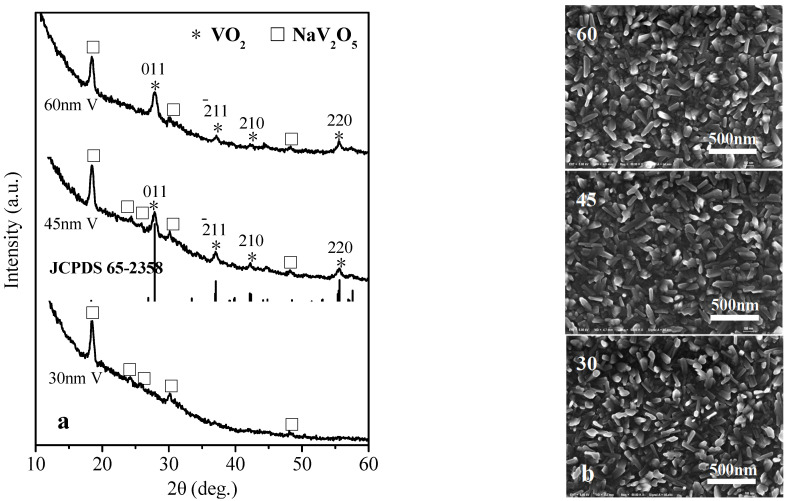
XRD patterns (**a**) and SEM images (**b**) of the VO_2_ thin films prepared by professional annealing of the 30, 45, 60 nm V metal films at 450 Pa, 400 °C for 1 h.

The XRD pattern in Figure 3a shows that the phase composition of VO_2_ films prepared by annealing of 30, 45, and 60 nm V film at 450 Pa. With the thickness increasing of V film, the concentration ratio of NaV_2_O_5_: VO_2_ decreases. Because of the low concentration and micro size of the VO_2_ particles, the VO_2_ film from 30 nm V presents nearly no corresponding diffraction peaks. The VO_2_ film prepared by annealing of 45 and 60 nm V film at 450 Pa includes not only a VO_2_, but also a NaV_2_O_5_ diffraction peak. The (011) and (−211) lattice planes of VO_2_ grow preferentially, and their diffraction peaks are stronger. The crystalline phase (M, monoclinic) of the VO_2_ film corresponds to the standard card JCPDS 65-2358. Moreover, the particle sizes of the VO_2_ films by 45 and 60 nm V are both about 15 nm, as calculated by Scherrer formula. The SEM image in Figure 3b suggests that the microstructure of VO_2_ films prepared by annealing of 30, 45, and 60 nm V film at 450 Pa is more or less the same. Take the VO_2_ film from 45 nm V for instance, where the microstructure is composed of long sheet crystals (length: 210 nm, width: 50 nm, thickness: 20 nm). It can be inferred that the long sheets are NaV_2_O_5_ crystals, which present obvious oriented growth. The same morphology of NaV_2_O_5_ crystals was reported by Mjejri et al. [33]. Smaller VO_2_ crystalline particles are scattered in the interspace of NaV_2_O_5_ crystals, which are difficult to observe due to the influence of the depth of field of the long sheet NaV_2_O_5_ crystals.

**Figure 4:**
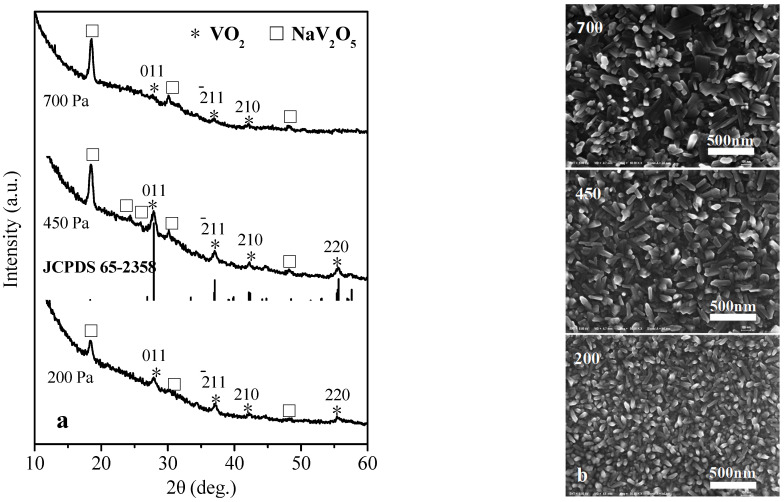
XRD patterns (**a**) and SEM images (**b**) of the VO_2_ thin films prepared by professional annealing of 45 nm V films at 200, 450, 700 Pa respectively, 400 °C for 1 h.

The XRD pattern in Figure 4a shows the phase composition of VO_2_ films prepared by the annealing of 45 nm V film at pressures 200, 450, 700 Pa. Compared to 200 Pa, the 450 and 700 Pa result in an obvious higher concentration of NaV_2_O_5_, and the 450 Pa leads to a higher ratio of VO_2_: NaV_2_O_5_ than the 700 Pa. Moreover, the VO_2_ film by 450 Pa performs crystalline phase (M) corresponding to the standard card JCPDS 65-2358. And the particle sizes of the VO_2_ films by 45 nm V at pressures 200, 450, and 700 Pa are about 12, 15, and 11 nm respectively, calculated by the Scherrer formula. The SEM image in Figure 4b suggests that the microstructures of VO_2_ films prepared by the annealing of 45 nm V film at pressures 200, 450, and 700 Pa are quite different. With the pressure increasing, the film microstructure presents bigger and bigger single crystals with an obvious oriented growth and a long sheet morphology, which are inferred to be NaV_2_O_5_ likewise. VO_2_ crystalline particles are scattered in the single crystals with depth of field and are difficult to observe.

**Figure 5:**
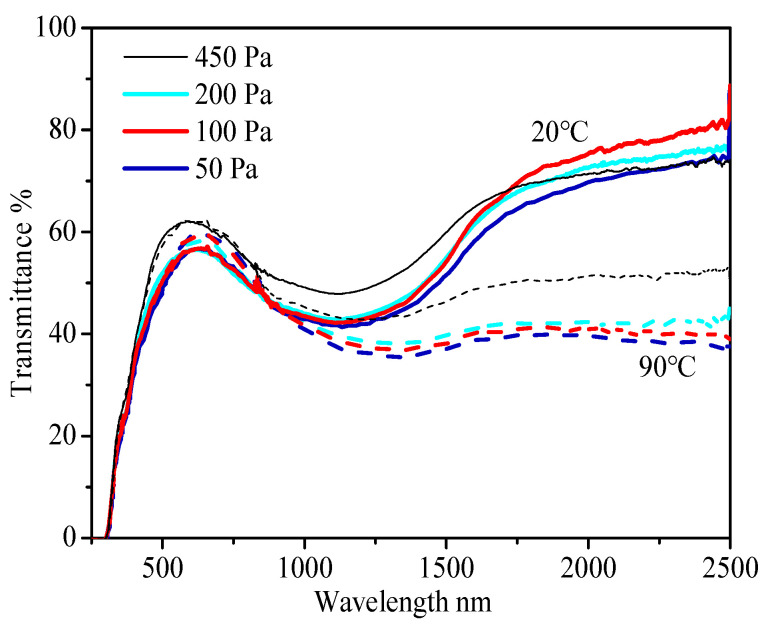
Transmittance of thinner VO_2_ films prepared by professional annealing of 30 nm V metal films at different pressures, 400 °C for 1 h; solid line: 20 °C, dashed line: 90 °C.

When the thickness of V film is reduced from 45 nm to 30 nm, the optimal annealing pressure is reduced to 100 Pa, as shown in Figure 5. In general, thicker V metal films require higher optimal annealing pressure to obtain large NIRSE, and thinner V metal films require lower optimal annealing pressure to obtain large NIRSE. The optical properties of the reduced VO_2_ thin films are shown in Table 5.

### 3.2. Transmittance, Loops and Microstructure of W-Doped VO_2_ Thin Films

**Figure 6:**
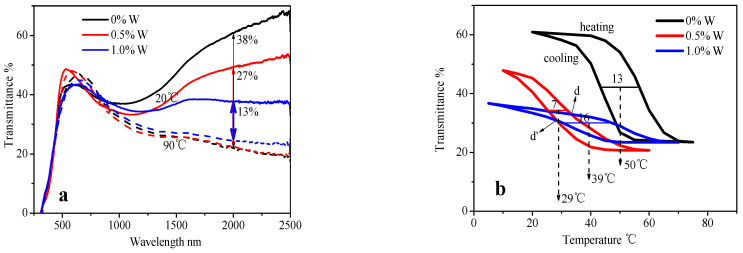
Transmittance curves (**a**) and thermal hysteresis loops (**b**) of VO_2_ films with different W doping concentrations prepared by professional annealing of 45 nm W:V films at 450 Pa, 400 °C for 1 h; solid line: 20 °C, dashed line: 90 °C in (**a**).

According to the transmittance curves in Figure 6a and the thermal hysteresis loops in Figure 6b of the W-doped VO_2_ films by 45 nm W:V at 450 Pa, the phase transition temperature (T_c_) of 0.5%W-doped VO_2_ thin film is significantly decreased (T_c_ = 29 °C). However, the NIRSE remains high (27%), and the shape of the thermal hysteresis loop is not distorted seriously. With the increase in the concentration of W dopant to 1% (i.e., 1.0%W-doped VO_2_ film), the phase transition temperature does not decline continuously but rises, especially above the temperature T_d_ or T_d′_ (33 °C or 28 °C), due to which the average T_c_ decreases to 39 °C. In addition, the NIRSE is negatively affected and reduces to 13%. At the same time, the shape of the thermal hysteresis loop is seriously distorted: the height decreases (13%, while for 0.5%W: 27%), the gradient becomes more gentle, and the width increases considerably (16 °C, while for 0.5%W: 7 °C).

**Figure 7:**
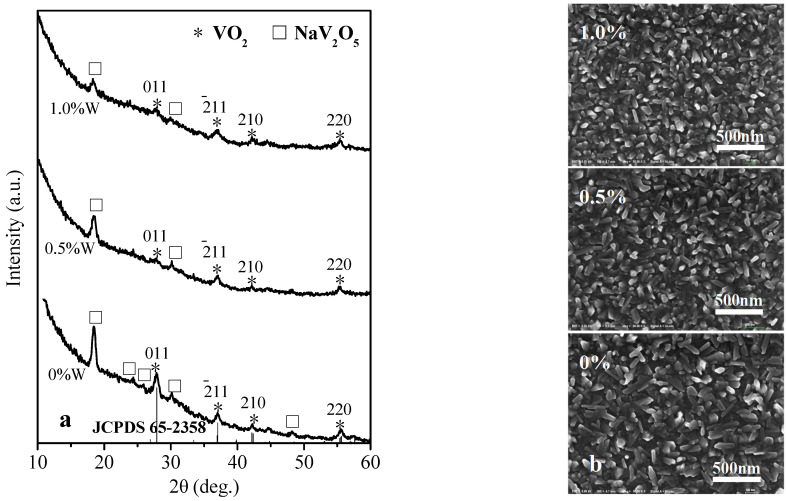
XRD patterns (**a**) and SEM images (**b**) of the W-doped VO_2_ thin films prepared by professional annealing of 45 nm W:V films at 450 Pa, 400 °C for 1 h.

The XRD pattern in Figure 7a shows that the peak intensities of the crystal NaV_2_O_5_ and the crystalline VO_2_ both decrease with the increase of W dopant. The SEM image in Figure 7b suggests that the size of the long sheet NaV_2_O_5_ crystal declines gradually with the increase of W dopant, which corresponds to the result of XRD. The W-doped VO_2_ crystallites cannot be seen in the SEM image for the depth of field of the long sheet NaV_2_O_5_ crystals, but the particle size calculated from XRD pattern by the Scherrer formula presents a decline trend too, with 15, 13, and 11 nm, corresponding to 0%, 0.5%, and 1.0%W respectively. W doping makes local T-VO_2_ phase increase and M-VO_2_ phase decrease, which results in tighter lattice structure and greater difficulty for Na diffusion into the film. In addition, W atom has more free electrons, which leads to a greater reducibility of the doped V film and higher consumption of oxygen. In conclusion, the above two reasons result in the decrement of the crystal NaV_2_O_5_ and the crystalline VO_2_.

### 3.3. Performance and Micro Morphology of VO_2_ Film on Intermediate SiO_2_ Layer

**Figure 8:**
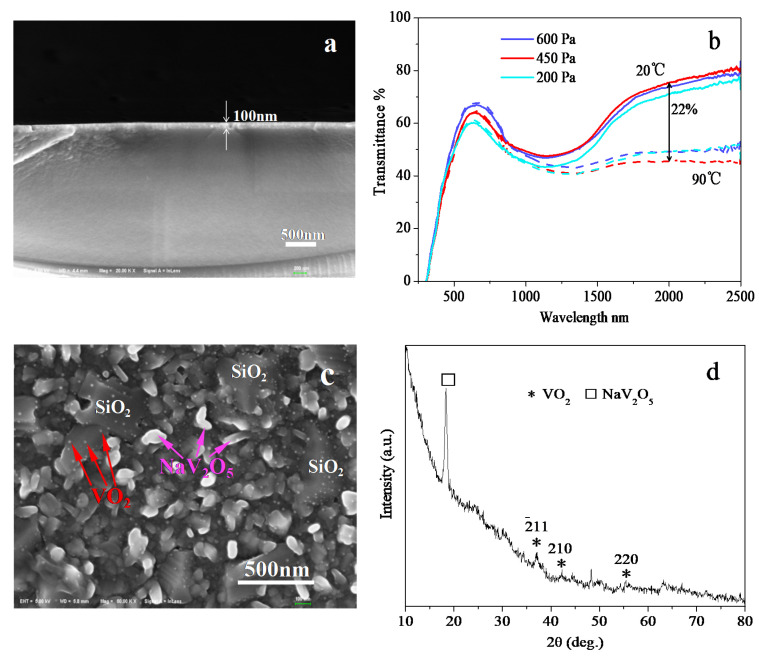
Cross-sectional SEM image of the intermediate SiO_2_ layer (**a**); transmittance curves of VO_2_ thin films on SiO_2_ prepared by professional annealing of 30 nm V at different pressures (**b**); SEM (**c**) and XRD (**d**) patterns of the VO_2_ thin film on SiO_2_ prepared by professional annealing of 30 nm V at 450 Pa; all at 400 °C for 1 h.

The thickness of the intermediate SiO_2_ layer prepared by RF magnetron sputtering is approximately 100 nm (Figure 8a). Then, 30 nm V film is deposited on it by DC magnetron sputtering (Table 1). Further, VO_2_ films are prepared by professional annealing of the V metal films at 200, 450, and 600 Pa (Table 3). According to the SEM image of the VO_2_ film prepared at the optimized pressure 450 Pa (Figure 8c), although the SiO_2_ layer is partly punctured, the number of long sheet crystals is significantly reduced. Moreover, it becomes easier to observe the VO_2_ crystallites (size about 14 nm by Scherrer Formula) scattering in the film (size about 14 nm too by SEM and software Nano Measurer 1.2.5). Compared with the VO_2_ film without SiO_2_ layer in Figure 3b-30 nm V, it indicates that the SiO_2_ layer plays a crucial role in blocking the diffusion of Na^+^ ions, which leads to an increase of the ratio of VO_2_: Na-V-O according to the XRD patterns in Figure 3a and Figure 8d, 30 nmV. Consequently, the NIRSE rises (22% at 450 Pa, Figure 8b; original: 20%, Figure 2a), and the near-infrared energy modulation ability △T_ir_ is also enhanced (8.8%; original: 7.0%). Meanwhile, the XRD pattern (Figure 8d) demonstrates that the 100 nm intermediate SiO_2_ layer cannot completely prevent the diffusion of Na^+^ ions, and a large number of NaV_2_O_5_ crystals are still generated due to pinholes in the SiO_2_ and diffusion of Na through it.

## 4. Discussion

### 4.1. Necessity of Two Equipments and Two Steps

The two-step method of magnetron sputtering-professional annealing used in this study not only has good repeatability, but also causes no heat damage to the magnetron sputtering equipment, which can prolong the service life of the equipment. Crystallization using special high-temperature equipment can promote a better phase transition performance. The method can be easily optimized to adjust the doping concentration, oxygen vacancies, content of high-valence NaV_2_O_5_, film thickness, etc., which are very useful to regulate the phase transition temperature, visible transmittance, and other thermochromic properties.

### 4.2. Effect of Substrate on the Preparation and Property of VO_2_ Thin Film

**Figure 9:**
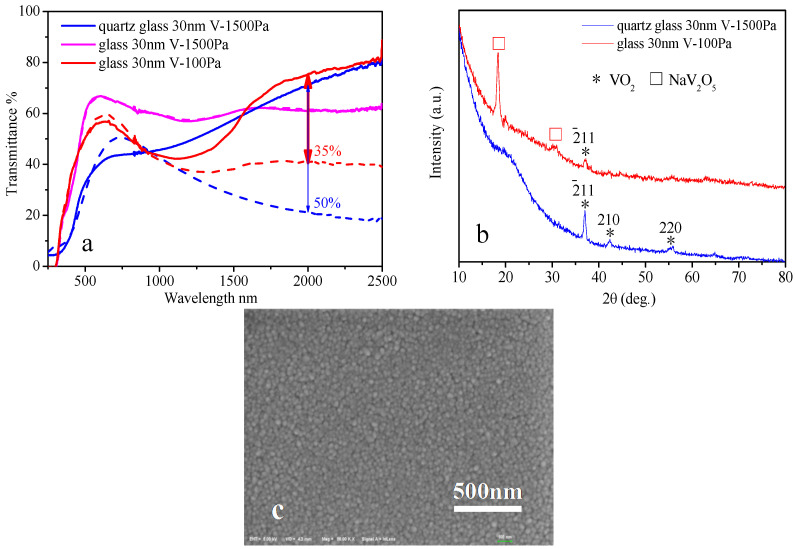
Transmittance curves (solid line: 20 °C, dash line: 90 °C) (**a**), XRD patterns (**b**) of VO_2_ films prepared by professional annealing of 30 nm V metal films on two contrastive substrates of quartz glass and float glass at set pressures, 400 °C for 1 h; and SEM image (**c**) of the VO_2_ film from “quartz glass 30 nm V-1500 Pa”.

Generally, the NIRSE of VO_2_ film on the glass substrate is lower than that on the quartz glass substrate (Figure 9a). This is because a part of the V atoms react with Na atoms from glass, O atoms from glass, and air to form NaV_2_O_5_, which results in low production of crystalline VO_2_, as shown in the XRD pattern (Figure 9b). In terms of the preparation technique, the VO_2_ film prepared on the quartz glass by annealing 30 nm V film at 1500 Pa exhibits a NIRSE value of 50% (Figure 9a). However, the film prepared on the glass by annealing 30 nm V film at 1500 Pa shows a NIRSE value of 0%. Nevertheless, when the annealing pressure is reduced to 100 Pa, a high NIRSE value (35%, Figure 9a) is achieved. The surface of VO_2_ film on quartz glass is flat and compact (Figure 9c), and like a passivation coating, can stand up to the high oxidation pressure 1500 Pa. Meanwhile, the surface of VO_2_ film on glass is a loose structure consisted of disordered long sheet NaV_2_O_5_ crystals and pinholes, which results in greater easiness of oxygen indiffusion from the atmosphere and easiness of oxidization of V to high valence NaV_2_O_5_ under a high pressure. In addition, there are more free non-bridged oxygen atoms in the glass substrate. Despite the much greater difficulty of O^2−^ ion outdiffusion than Na^+^ for its strong Coulomb force, there still is the possibility of O emigrating to the surface and oxidizing the V metal film during the annealing. However, in quartz glass, O atoms are firmly bonded with Si atoms by the covalent bonds and are very difficult to move. So, in conclusion, when on glass substrate, the oxidation pressure is much lower, because of the loose surface of the film majorly, as well as the O outdiffusion from the substrate minorly; and when on quartz glass, the oxidation pressure could be much higher, because of the compact surface of the film.

### 4.3. Choice between NIRSE and T_lum_

The primary goal of VO_2_ film preparation is to maximize the value of NIRSE, which means that the content and purity of VO_2_ in the film should reach the highest level. However, for smart windows, both NIRSE and visible transmittance T_lum_ should be considered. The minimum daylighting rate of green building glass is generally required to be above 60%. Therefore, it is useful to sacrifice a part of NIRSE to improve the T_lum_, while ensuring an appropriate high value of NIRSE. The common method to improve the visible transmittance is to deposit anti-reflection films. However, two other simple approaches were used in this study: thinning the V metal film and increasing the annealing pressure. For instance, when the V metal film is thinned from 45 nm to 30 nm, the NIRSE decreases from 39% to 20%, but the T_lum_ increases from 44.5% to 63%, as shown in Figure 2a and Table 4. When the annealing pressure of 45 nm V film increases from 450 Pa to 700 Pa, the NIRSE decreases from 39% to 30%, but the T_lum_ increases from 44.5% to 61%, as shown in Figure 2b and Table 4. The two methods’ mechanism is: at the same high enough oxidation pressure, thinner V metal films will be more easily oxidized into high-valence vanadium; and for the V metal films with same thickness, higher oxidation pressure will result in more high-valence vanadium likewise. The content of low-valence vanadium (mainly crystalline VO_2_) in the film decreases, and the content of high-valence vanadium (mainly crystal NaV_2_O_5_) increases, which leads to an increase in the optical band gap, and results in the increase of the visible transmittance of the film. Moreover, there is another for the thinner film reason, namely lower light absorption.

### 4.4. Mechanism of W Doping and Equivalent Effect of O Vacancy

There are two mechanisms through which W doping reduces the phase transition temperature (T_c_) of VO_2_: Peierls mechanism and Mott mechanism. Among them, the Peierls mechanism is described by the energy band theory [34,35,36], where structural change is considered as the driving force. After larger W atoms replace the smaller V atoms, the lattice expands and the V-V dimerization pairs are destroyed, which results in the change of energy band position. The band gap between V3d_//_and π* becomes narrower, the electronic migration becomes easier, and the phase transition barrier is reduced, so the T_c_ decreases. In addition, according to the energy band theory, T_c_ continues to decline with the increase in the concentration of W dopant, which disagrees with our experimental results (decreases first and then rises). Therefore, this paper supports the second theory of the Mott mechanism. The Mott mechanism is described by the charge-doping theory [37,38,39], where electron correlation is the driving force. After V^4+^ ions are replaced by W^6+^ ions, the excess electrons enter the empty π* band, and then local conduction bands and local metallic phases are formed. Therefore, the transformation into tetragonal phase becomes easier, and thus the transition temperature T_c_ declines. However, excessive W doping can cause superabundant electrons to enter the empty π* band, and the interaction of electrons is enhanced, which results in the splitting of π* band and the formation of a new band gap. The phase transition barrier becomes larger, and thus the T_c_ rises again. The mechanism of W doping to reduce the phase transition temperature of VO_2_ is still under debate, just like the debate between Peierls mechanism and Mott mechanism for the intrinsic phase transition of pure VO_2_ [40,41]. Presently, there is no unified theory that can fully describe the effect of W doping.

Although the phase transition temperature is reduced by W doping, the thermochromic properties are adversely affected, and the most significant change is that the NIRSE declines significantly. The local conduction bands are formed after W doping, and the electronic transition becomes easier. At low temperature, the ability of reflecting infrared radiation is enhanced, and the infrared transmittance is reduced, and thus the NIRSE is clearly reduced. With the increase of W dopant, the thermal hysteresis loop becomes gentle (Figure 6b) and is no longer steep. This is directly related to the reduction of phase transition temperature at low temperature (below T_d_ or T_d’_), the rise of phase transition temperature at high temperature (above T_d_ or T_d’_), and the decline of NIRSE. At low temperature (below T_d_ or T_d’_), due to the increase of W dopant, local conduction bands in empty π* band are increased, and the monoclinic-tetragonal phase transition can be easily realized, which results in the reduction of the phase transition temperature. At high temperature (above T_d_ or T_d’_), due to the gradual increase of tetragonal metallic phase, more and more electrons enter the empty π* band, and the interaction becomes increasingly stronger, which results in worse fragmentation of the π* band, a wider band gap, larger phase transition barrier, and thus the increase of the phase transition temperature.

**Figure 10:**
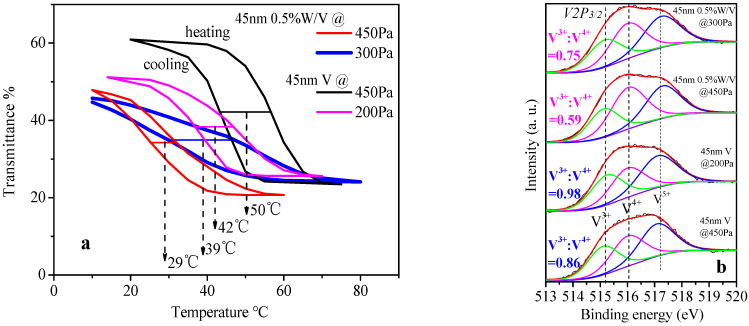
(**a**) Effect of lowering the annealing pressure on the thermal hysteresis loop of the prepared VO_2_ films and W/VO_2_ films (from 450 Pa to 200 Pa for 45 nm V; from 450 Pa to 300 Pa for 45 nm 0.5%W/V; all at 400 °C for 1 h); (**b**) XPS spectra of V^3+^, V^4+^ of the corresponding VO_2_ films and W/VO_2_ films.

Similar to W doping, the oxygen vacancies can lower the phase transition temperature of VO_2_, which were created by lowering the annealing pressure. For example, the T_c_ of VO_2_ film fabricated by annealing 45 nm V film at 200 Pa is 8 °C lower than that of VO_2_ film fabricated by annealing 45 nm V film at 450 Pa, as shown in Figure 10a. The oxygen vacancies lead to the transformation of V^4+^ into V^3+^ and generation of excess electrons, which enter the empty π* band and form a local conduction band. The growth of the charge carrier concentration and the enhancement of the metal phase results in the decline of phase transition temperature, which is similar to the effect of W doping at low concentration. However, it should be noted that when W dopant is used for lowering the phase transition temperature of VO_2_, and the concentration has reached an optimal value, i.e., a level (such as 0.5%W) that can cause T_c_ to rise rather than continually decline, the preparation of oxygen vacancies has an opposite effect. For instance, the T_c_ of the VO_2_ film prepared by annealing 45 nm 0.5%W/V film at 300 Pa is 10 °C higher than that of the VO_2_ film prepared by annealing 45 nm 0.5%W/V film at 450 Pa, as shown in Figure 10a. The formation of oxygen vacancies is indicated by the increase of the ratio of V^3+^: V^4+^ in the V2p_3/2_ XPS spectra (Figure 10b). The three fitting peaks correspond to V^3+^ (515.2 eV), V^4+^ (516.0 eV), and V^5+^ (517.2 eV), respectively [42]. It is found that the V^3+^: V^4+^ ratio increases from 0.86 to 0.98 when the annealing pressure decreases from 450 Pa to 200 Pa for 45 nm V, and increases from 0.59 to 0.75 when the annealing pressure decreases from 450 Pa to 300 Pa for 45 nm 0.5%W/V.

In addition, the causes of the decrement of T_c_ should probably include the strain in the film, as studied by Elia [43], Paez [44], and so on [45]. The substrate used her is glass, so there would not be induction for strain generation by certain lattice planes such as TiO_2_(001) [43,44,45]. However, in spite of this, there would be some strain inevitably existing in the interface between glass and W/VO_2_ film, grain boundaries, lattice deformation, and so on. So, the strain in the film after W doping would be another cause for T_c_ reduction, though not the main cause.

### 4.5. Effect of 100 nm Intermediate SiO_2_ Layer

As described in Section 3.3, a 100-nm-thick intermediate SiO_2_ layer pre-deposited on the glass plays an obvious role in blocking the diffusion of Na^+^ ions. However, it does not stop the diffusion completely, and many NaV_2_O_5_ crystals are still generated. As a next step, increasing the thickness of the intermediate SiO_2_ layer or replacing it with a better layer (compact, hard, narrow lattice plane, etc.) can completely block the diffusion of Na^+^ ions. Meanwhile, the addition of the intermediate SiO_2_ layer changes the technical parameters for the preparation of VO_2_ thin film, and the optimal oxidation pressure increases from 100 Pa to 450 Pa (Figure 5 and Figure 8b). Similar to the mechanism described in Section 4.2, the intermediate SiO_2_ layer causes fewer long sheet NaV_2_O_5_ crystals and a flatter, more compact surface than that on glass substrate (Figure 3b-30 nm V, Figure 8c), which results in the difficulty of oxygen indiffusion from the atmosphere. Besides, the intermediate SiO_2_ blocks the migration of non-bridged oxygen atoms from the glass into the film. In conclusion, the demand of oxidation annealing pressure rises, mainly because of the compacter surface of the film, and minorly because of the block of O immigration into film from glass.

## 5. Conclusions

Magnetron sputtering and the professional annealing method have the advantages of simple operation, convenient optimization, good repeatability, and no heat damage to the sputtering machine. Therefore, the represent an appropriate method for the fabrication and systematical examination of VO_2_ thin films. To obtain the largest NIRSE value is the primary objective. The thickness must match with the optimal pressure. Otherwise, the value of NIRSE will decline. Under the fixed annealing condition of 400 °C (1 h), a thicker V-based metal film requires a higher optimal annealing pressure, and the thinner film requires a lower optimal annealing pressure. When a glass substrate is used, mainly because of the loose surface structure of disordered long sheet NaV_2_O_5_ and pinholes, the oxidation annealing pressure reduces sharply. In this study, it was verified that compared with the quartz glass substrate, the annealing pressure of the 30 nm V metal film on the glass substrate decreased by more than 90%. Increasing the annealing pressure or decreasing the thickness of V metal film was demonstrated as a new effective method to improve the visible transmittance T_lum_. The 100 nm intermediate SiO_2_ layer played an obvious role in preventing the diffusion of Na^+^ ions, though not completely. Thicker or new intermediate layers need to be further studied. This study supports the charge-doping theory. A proper concentration of W dopant can lead to the formation of local conduction bands in the π* band, which can reduce the T_c_ of VO_2_ thin film. However, excessive W doping can lead to the splitting of π* band, which results in the formation of a new band gap and an increase in the phase transition barrier, and thus the T_c_ increases. In terms of lowering the T_c_ of VO_2_ film, oxygen vacancy and W dopant have an equivalent effect. Further, when the W doping concentration is optimal, no more oxygen vacancies should be added. Otherwise, an opposite effect can occur.

## Figures and Tables

**Table 1:** Preparation parameters of V-based metal films by magnetron sputtering.

Substrate	Target	Background Pressure (Pa)	Working Gas	Working Pressure (Pa)	Substrate Temperature (°C)	Sputtering Method	Power (W)	Sputtering Time (min)	V Film Thickness (nm)
Glass	V	3.0 × 10^−3^	Ar	1	20	DC	77	5, 7.5, 10	30, 45, 60
Quartz glass	V	3.0 × 10^−3^	Ar	1	20	DC	77	5	30
SiO_2_ (/glass)	V	3.0 × 10^−3^	Ar	1	20	DC	77	5	30
Glass	W/V	3.0 × 10^−3^	Ar	1	20	DC	77	7.5	45

**Table 2:** Preparation parameters of the intermediate SiO_2_ layer by RF magnetron sputtering.

Substrate	Target	Background Pressure (Pa)	Working Pressure (Pa, Ar)	Substrate Temperature (°C)	Sputtering Method	Power (W)	Sputtering Time (min)	SiO_2_ Film Thickness (nm)
Glass	SiO_2_	3.0 × 10^−3^	1	200	RF	100	50	100

**Table 3:** Preparation parameters of VO_2_-based films by professional annealing.

Metal Film/Substrate	Annealing Atmosphere	Background Pressure (Pa)	Annealing Temperature (°C)	Heating Rate (°C/min)	Annealing Time (h)
30 nm V/glass	air	50, 100, 200, 450, 1500	400	5	1
45 nm V/glass	air	200, 450, 700	400	5	1
60 nm V/glass	air	450	400	5	1
30 nm V/quartz glass	air	1500	400	5	1
30 nm V/SiO_2_/glass	air	200, 450, 600	400	5	1
45 nm W:V/glass	air	300, 450	400	5	1

**Table 4:** Optical properties of VO_2_ thin films prepared by professional annealing under optimized thickness and pressure.

V Film Thickness (nm)-Annealing Pressure (Pa)	NIRSE (@2000 nm)	ΔT_ir_ (780–2500 nm)	T_lum_ (20 °C)	T_lum_ (90 °C)	T_lum_ (Average)
30–450	20%	7.0%	63%	63%	63%
45–450	39%	10.9%	44%	45%	44.5%
60–450	26%	10.1%	41%	38%	39.5%
45–200	16%	7.4%	43%	44%	43.5%
45–700	30%	4.2%	61%	61%	61%

**Table 5:** Optical properties of thin VO_2_ films prepared by 30 nm V metal films at different annealing pressures.

Annealing Pressure (Pa)	NIRSE (@2000 nm)	ΔT_ir_ (780–2500 nm)	T_lum_ (20 °C)	T_lum_ (90 °C)	T_lum_ (Average)
450	20%	7.0%	63%	63%	63%
200	30%	7.2%	57%	57%	57%
100	35%	8.0%	56%	58%	57%
50	30%	7.3%	55%	57%	56%

## Data Availability

The data presented in this study are available on request from the corresponding author.
